# Commentary on two reports on animal models of COVID‐19

**DOI:** 10.1002/ame2.12127

**Published:** 2020-06-24

**Authors:** Cynthia Pekow

**Affiliations:** ^1^ International Council for Laboratory Animal Science Brussels Belgium

In this communication, I have been invited to comment on the significance of two recent reports: The Pathogenicity of 2019 Novel Coronavirus in hACE2 Transgenic Mice^1^ and Age‐related Rhesus Macaque Models of COVID‐19.^2^ The research was performed under the auspices of the Chinese Academy of Medical Sciences and Comparative Medicine Center, at Peking Union Medical College in Beijing, China. I am a Laboratory Animal Veterinarian, currently serving as the President of the International Council for Laboratory Animal Science (ICLAS). I have no affiliation or conflicting interest to report. The opinion expressed here is my own.

In the fear and uncertainty of the COVID‐19 pandemic, we look to science for reliable methods for diagnosis, treatment, prevention, and cure. Development of these methods requires understanding many facets of this new coronavirus, from how it is spread, to which species it infects, to the pathology it causes. We seek to know how COVID‐19 differs from similar coronaviruses such SARS (2002) and MERS (2012), and why this virus is particularly, but not exclusively, more dangerous for older individuals. We need to learn why some patients’ immune or clotting systems are spurred to lethal over‐reaction while fighting COVID‐19 infection. We must ascertain the course of antibody production induced by this viral infection, and whether protection from re‐infection is conferred, and for how long. As with SARS and MERS, animal models will provide an essential means to investigating these and other urgent questions about this new coronavirus.

Mice are not a natural host for COVID‐19. However, the ability to manipulate the mouse genome allows creation of animals susceptible to infection by specific infectious agents. Thanks to their rapid reproduction and large litters, small size, relative ease of housing and containment, plus in‐depth knowledge of their biology, mice are a natural choice to help understand COVID‐19 infection in a mammalian system. In the report posted in bioRxiv, the research team led by Qin Chuan built upon knowledge gained from a mouse model previously proven susceptible to the genetically related coronavirus that causes SARS. The human Angiotensin‐converting enzyme 2 (hACE2) receptor is necessary for the SARS virus to gain cell entry. Mice were engineered to express hACE2 receptors on airway epithelial cells and epithelia of other internal organs. This study was one of the firsts to establish that this mouse model is also susceptible to infection with COVID‐19. In this report, the same receptor facilitated the entry of the COVID‐19 virus into the animal cells. The pathology described in the mice challenged with COVID‐19 included interstitial hyperplasia in lung tissue, and some inflammation in the bronchioles and blood vessels. The histology post infection is consistent with what would be seen with viral pneumonia. Animals challenged with the virus but lacking the ACE2 receptors in the lungs did not show this pathology. Virus isolated from infected animals was shown to infect cells in culture.

The main limitations of this report are that numbers of animals are low, and additional information is needed on the genetic background of the control animals and microbial status. The genetic background of the mice may influence the immune response, for example, in T helper cell populations (Th1 and Th2), which can vary between inbred mouse strains.^3^ However, this preliminary study is of significance in showing that this transgenic mouse has great potential for use as a model to understand a number of key questions about the new coronavirus and its pathology. Mice with the hACE2 transgene are commercially available, on different genetic backgrounds; commercial suppliers are breeding these animals to meet the sudden need. COVID‐19 infection of the hACE2 transgenic mouse in particular may prove useful as a model for vaccine development.

In the second paper, a description is made of a model of COVID‐19 infection in older (15 years) and young (3‐5 years) rhesus macaques. The significant advantage of a non‐human primate model of infection is of course their similarity to man, and the great likelihood of translation to the human condition from what is learned in this model. Scientists must carefully parse the use of this precious resource; research with non‐human primates, and particularly with infectious agents in these animals, requires highly specialized centers and personnel to contain the infection and work safely and humanely with these intelligent animals. The report describes an important and prudent first step in establishing this primate model, using limited numbers of animals.

Worldwide, the greatest percentage of deaths caused by COVID‐19 is in the population aged 45 and older, with particularly high death rates in the oldest age groups. Infection in younger people, and particularly in children, is often asymptomatic, and there is limited understanding of the role of people with asymptomatic infection in spreading the virus to others. Control methods to rein in the pandemic will be guided by the knowledge of how people of different age groups react to the virus, and how and for how long they may shed the virus. Rhesus macaques age at about 3 times the rate at which humans age. Puberty occurs between 2.5 and 4.5 years, and lifespan is about 27 years, with a maximum of about 40 years. Macaques and humans mature in a similar fashion, and are very much alike in anatomy, metabolism, endocrine and immune systems, reproduction, and aging.^4^

This study compared the course of infection over 14 days of a young cohort of three animals (ages 3‐5 years) with an older pair (15 years of age) in their response to intratracheal inoculation with COVID‐19. The study provides an intriguing preliminary look at what appears to be an excellent model for study of COVID‐19. The results point to a more severe course of injury in the older animals, with fewer signs of pathology in the younger cohort. An encouraging sign is the antibody response seen in both age groups by 14 days after the infection was initiated. Pathology results with supporting histology show the older cohort were most severely affected by the infection, mirroring what is seen in human infections with this coronavirus.

Many answers to the puzzles posed by COVID‐19 will come with time, with trial and error, and alas after many deaths across the globe. But for many of the urgent questions on disease spread, diagnosis, treatment, and immunity we can and must look to animal models to further our understanding rapidly. These animal models can hasten the development of better diagnostics, treatments, preventions, and cures.^5^ The initial investigations into establishing new animal models led by Chuan Qin and reported by Linlin Bao et al and by Pin Yu et al, are an important step forward. These studies reflect a well‐considered and positive start to establishing necessary animal models to help us find the vital strategies to control the COVID‐19 pandemic. I am grateful to these researchers for leading early and crucial steps in establishing what are likely to be key resources for scientists across the world working to solve the mysteries of the new virus.

## CONFLICT OF INTEREST

None.
